# Analyzing Respiratory Sinus Arrhythmia: A Markov Chain Approach with Hypertensive Patients and Arachnophobic Individuals

**DOI:** 10.3390/muscles3020016

**Published:** 2024-06-20

**Authors:** Erika Elizabeth Rodriguez-Torres, María Fernanda Azpeitia-Cruz, Jaqueline Escamilla-Muñoz, Isaac Vázquez-Mendoza

**Affiliations:** Área Académica de Matemáticas y Física, Universidad Autónoma del Estado de Hidalgo, Pachuca 42184, Mexico; az335194@uaeh.edu.mx (M.F.A.-C.); es419646@uaeh.edu.mx (J.E.-M.); va296000@uaeh.edu.mx (I.V.-M.)

**Keywords:** Markov chain model, respiratory sinus arrhythmia, arachnophobia, RR intervals, heart rate variability (HRV), electrocardiogram (EKG)

## Abstract

Respiratory Sinus Arrhythmia (RSA) manifests as cyclic fluctuations in heart rate in synchrony with breathing. Gaining insights into the dynamics of RSA within the cardiac muscle functioning is crucial for comprehending its physiological and clinical significance. This study presents an analytical framework employing Markov chains to probe RSA patterns, with a specific emphasis on individuals with hypertension and arachnophobia. We delve into the concept of RSA and its potential cardiovascular implications, particularly among populations characterized by hypertension or normotension and fear of spiders. This study utilizes Markov chain modeling, an innovative method used to scrutinize RSA dynamics across diverse cohorts, with the aim of unveiling underlying patterns and mechanisms. This research contributes to the advancement of our understanding of RSA and its correlation with cardiac function across varied demographics, potentially guiding tailored diagnostic and therapeutic interventions. Our findings highlight significant disparities between hypertensive and normotensive participants, as well as spider-fearful individuals employing techniques to confront their fear compared with those without such strategies.

## 1. Introduction

In the context of analyzing Respiratory Sinus Arrhythmia (RSA) in the cardiac muscle functioning, Markov chains offer a powerful tool for modeling and understanding the dynamic behavior of heart rate variability (HRV), according to Simpson et al., 2022 [1]. Representing different states of HRV as discrete states in a Markov chain, e.g., high and low variability, the probabilities associated with transitioning between these categorical states over time can be elicited.

We hypothesized that Markov chains offer a powerful and versatile approach for analyzing RSA in cardiac muscle for hypertensive patients and also for arachnophobic individuals given that when individuals with a specific phobia are triggered, they experience fear and anxiety responses, which can lead to sudden changes in heart rate variability [2]. By leveraging the inherent temporal dependencies in heart rate variability data, Markov chain models provide critical insights into autonomic regulation, cardiac function, and cardiovascular health, ultimately advancing our understanding of RSA dynamics and its clinical significance.

### 1.1. Markov Chains

One of the crucial advantages of using Markov chains is their ability to capture temporal dependencies in sequential data. In the case of RSA analysis, Markov chains can account for the sequential nature of heart rate fluctuations and capture how the current state of heart rate variability influences future states. This enables the identification of patterns and trends in RSA dynamics and the gaining of insights into underlying physiological mechanisms.

Furthermore, Markov chains provide a flexible framework for analyzing complex systems with multiple interacting components [3]. In the context of RSA analysis, Markov chain models can incorporate additional variables such as respiratory rate, sympathetic and parasympathetic activity, and other physiological parameters to provide a more comprehensive understanding of autonomic regulation and cardiac function [4].

By applying Markov chain modeling techniques to RSA data, valuable information about the underlying dynamics of cardiac autonomic control and its relationship to overall cardiovascular health can be extracted. This has important implications for both basic research and clinical practice, including the development of novel diagnostic tools, prognostic indicators, and personalized treatment strategies for cardiovascular diseases.

### 1.2. Cardiac Muscle

The heart is a remarkable organ composed predominantly of muscle tissue known as cardiac muscle or myocardium. This specialized muscle type possesses unique characteristics that enable the heart to perform its vital function of pumping blood throughout the body.

The cardiac muscle is arranged in a highly organized structure, consisting of individual cells called cardiomyocytes. These cells are elongated and branched, forming interconnected networks that facilitate coordinated contraction. Intercalated discs and specialized structures between cardiomyocytes provide mechanical and electrical coupling, ensuring synchronous contraction of the heart muscle [5].

The primary function of the cardiac muscle is to contract rhythmically, generating the force necessary to propel blood through the circulatory system. Unlike the skeletal muscle, which requires external stimulation from nerves to initiate contraction, the cardiac muscle exhibits intrinsic rhythmicity due to the presence of specialized pacemaker cells in the sinoatrial node. This inherent ability for spontaneous contraction allows the heart to maintain a consistent heartbeat, essential for sustaining life [6].

Cardiac muscle contraction is regulated by a complex interplay of electrical and biochemical signals. The heartbeat is initiated by electrical impulses generated in the sinoatrial node, propagating through the heart via specialized conducting fibers. These impulses trigger calcium release from intracellular stores, leading to actin–myosin cross-bridge formation and subsequent muscle contraction. The duration and strength of contraction are finely regulated by various neurotransmitters, hormones, and ion channels, ensuring optimal cardiac function under diverse physiological conditions [5].

The cardiac muscle exhibits remarkable adaptability in response to changing physiological demands. Through a process known as cardiac remodeling, the heart can adjust its size, shape, and contractile properties in response to factors such as exercise, stress, and disease. While acute increases in workload can lead to physiological hypertrophy, chronic stressors may result in pathological remodeling, characterized by structural and functional abnormalities that impair cardiac function [7].

Understanding the structure and function of the cardiac muscle is crucial for diagnosing and treating a wide range of cardiovascular disorders. Conditions such as hypertension, stressful events (arachnophobia), myocardial infarction, heart failure, and arrhythmia are often associated with abnormalities in cardiac muscle structure or function. Advances in cardiac imaging techniques and molecular biology have facilitated the development of innovative therapies to preserve or restore cardiac muscle function, i.e., enhancing cardiac muscle resilience, expecting improved outcomes for patients with heart diseases [8].

### 1.3. Respiratory Sinus Arrhythmia

Respiratory Sinus Arrhythmia (RSA) is a natural phenomenon characterized by rhythmic variations in heart rate that synchronize with the respiratory cycle [9]. The term *sinus* refers to the variations originated by the sinoatrial (SA) node, the heart’s natural pacemaker. During inspiration, when air is drawn into the lungs, the heart rate typically increases, while during expiration, when air is expelled from the lungs, the heart rate decreases  [10]. This cyclical variation in heart rate is due to complex interactions between the autonomic nervous system and cardiac pacemaker cells.

The primary mechanism underlying RSA involves the interplay between the parasympathetic and sympathetic branches of the autonomic nervous system [11]. During inhalation, an increased activity of the sympathetic nervous system is observed, leading to the release of norepinephrine, which accelerates the heart rate. Conversely, during exhalation, parasympathetic activity predominates, leading to the release of acetylcholine, which slows down the heart rate. These opposing autonomic influences create the characteristic sinusoidal pattern observed in RSA.

RSA may be observed in individuals of all ages, from infants to elders, and is particularly prominent during the relaxation and sleep stages. It is considered a marker of cardiac autonomic function and is often used as an indicator of cardiovascular health [12]. Previous works have shown that alterations in RSA patterns may be associated with various physiological and pathological conditions, including cardiovascular diseases, respiratory disorders, and neurological conditions [13].

In addition to its physiological significance, RSA has garnered attention in clinical settings as a potential diagnostic tool. The analysis of RSA patterns, through either electrocardiography (EKG) or other physiological monitoring techniques, provides valuable insights into autonomic function and cardiovascular regulation [9]. Furthermore, abnormalities in RSA patterns may serve as early indicators of underlying health issues, prompting further investigation and intervention.

Understanding the background and mechanisms of RSA is essential for interpreting its significance in both physiological and clinical contexts. Continued research into RSA dynamics and its relationship to overall health and disease states promises to advance our understanding of cardiovascular function and improve diagnostic and therapeutic approaches in cardiology and related fields.

### 1.4. On the Construction of a Markov Chain

A Markov chain, named after the Russian mathematician Andrey Markov, is a powerful mathematical model utilized across diverse disciplines such as physics, biology, finance, and computer science. It describes a sequence of events where the probability of transitioning from one state to another solely depends on the current state and is independent of the events that preceded it [3]. This stochastic model provides a framework for analyzing complex systems by representing transitions between states probabilistically, enabling insights into dynamic processes without considering the entire history of events [14].

Let $X_{0},X_{1},X_{2},\ldots$ be a sequence of random variables representing the states of the system at discrete time points t=0,1,2,…. Each random variable $X_{t}$ takes values on a finite or countably infinite number of possible states.

The probability of transitioning from state *i* to state *j* at time t+1 given that the system is currently in state *i* at time *t* is denoted by $P_{ij}\left(t\right)$, where $P_{ij}\left(t\right)=P\left(X_{t+1}=j\;|\;X_{t}=i\right)$. These transition probabilities satisfy the following Markov property:

$$P\left(X_{t+1}=j\;|\;X_{t}=i,X_{t-1}=i_{t-1},\ldots ,X_{0}=i_{0}\right)=P\left(X_{t+1}=j\;|\;X_{t}=i\right)$$

for all t≥0 and all $i,i_{t-1},\ldots ,i_{0},j$ in the state space.

The collection of transition probabilities can be represented by a transition probability matrix *P*, where each element $P_{ij}$ represents the probability of transitioning from state *i* to state *j*. The *i*-th row of the matrix corresponds to the probabilities of transitioning from state *i* to all other states.

$$P=\left[\begin{array}{cccc} P_{00} & P_{01} & \cdots & P_{0n}\\ P_{10} & P_{11} & \cdots & P_{1n}\\ \vdots & \vdots & \ddots & \vdots \\ P_{n0} & P_{n1} & \cdots & P_{nn} \end{array}\right]$$

## 2. Materials and Methods

Two different studies were analyzed: One involves hypertensive and normotensive individuals, a work developed by Rojas-Vite et al., 2022 [15]. The other study involving participants regarded as spider-fearful individuals, herein referred to as the Arachnophobic group, is the work of Ihmig et al., 2020 [16].

### 2.1. Hypertensive Group

The study protocol received approval from the ethics and research committee of the General Hospital of Pachuca de Soto, Hidalgo (HGDP), under protocol number 2018/025. The research involved a review of the clinical files of individuals diagnosed with arterial hypertension, specifically selecting those patients with essential hypertension. The five participants selected were reported to have pharmacologically controlled high blood pressure (HBP), use medications such as telmisartan and losartan for over a year, and be in the age range between 35 and 55 years old. These patients had an average body mass index (BMI) of 28.35 ± 2.02. Additionally, their blood pressure had a systolic blood pressure (SBP) of 134.62 ± 11.3 and a diastolic blood pressure (DBP) of 89.5 ± 7.81. Individuals with concurrent conditions like obesity, diabetes, and heart or respiratory diseases were excluded from the study [15].

### 2.2. Normotensive Group

The control group consisted of five volunteers with a blood pressure below 120/80 mmHg and no reported diseases and within the same age range as the hypertensive group. The anthropometric indexes and blood pressure of this group were reported with an average BMI of 26.18 ± 2.99, an average SBP of 107.37 ± 10.33, and an average DBP of 72.87 ± 5.35 [15].

Prior to this study, participants of both groups (hypertensive and normotensive group) were instructed to abstain from consuming stimulant drinks or alcohol during the day before taking the EKG registers scheduled for assessment at 8:00 a.m. in the psychophysiology laboratory. All participants provided informed consent to participate in the study. The clinical evaluation included height and weight measurements for each participant, followed by a 5 min rest period in a seated position, after which blood pressure was measured on the left arm. Blood pressure was measured on the left arm due to the hospital’s accepted protocol, and it is only measured on the same arm for all patients. Subsequently, participants remained at rest, in silence, with their eyes open, while 30 min electrocardiographic (EKG) activity was recorded. The electrode placement for EKG recordings followed the protocol described by Shaffer and Ginsberg, 2017 [17]. For this study, the records of five hypertensive patients and five normotensive patients are used.

### 2.3. Arachnophobic Groups

The study comprised 57 participants whose ages ranged from 18 to 40 years old, with biological signals collected from July 2017 to July 2018 at the University of Saarland located in Germany. Participants are regarded as spider-fearful individuals (SFs) when they score 14 points or higher in the German Spider Anxiety Screening (SAS), which is the commonly used cut-off score. Moreover, they have to score 50 points or higher on the Fear of Spiders Questionnaire (FSQ). Additionally, SFs must reach a minimum fear score of 4 and an avoidance score of 3 in the section “Specific Phobias” of the structured interview for mental disorders (ADIS—section “Specific Phobias”). The exclusion criteria are the presence of any mental disorder other than spider phobia (assessed with the Patient Health Questionnaire D (PHQ-D) and the Beck Depression Inventory (BDI-II) and any reported cardiovascular disease (assessed during the screening phase). A description of the mentioned tests can be found in [18,19,20,21,22]. During the sessions, all subjects were asked to watch a series of spider video clips. Each session began with a 1 min demonstration clip, followed by 16 1 min spider video clips sourced from TV documentaries featuring detailed footage of spiders. The session concluded with a 5 min break phase. The 16 spider clips were divided into two groups: clips 1–8 and clips 9–16, with the order randomized within each group. Biological signals were recorded using the portable BITalino biosignal measurement device (PLUX Wireless Biosignals SA, Lisbon, Portugal) with the sampling frequency set at 100 Hz per channel and 10-bit resolution. For further information, refer to the work of Ihmig et al., 2020 [16].

All participants received a session during which they were assigned two tasks: HRV biofeedback and a motor pseudo-biofeedback task, or two motor pseudo-biofeedback tasks. Subsequently, participants were stratified into four distinct groups, each undergoing specific training protocols. Group 1 engaged in HRV biofeedback training alongside a motor pseudo-task before exposure, with continued HRV biofeedback use during the clip exposure. Group 2 followed the same regimen as Group 1 but maintained the pseudo-biofeedback task during exposure. Group 3 underwent training in two pseudo-biofeedback tasks, carrying forward only one during exposure. Lastly, Group 4 received training in two pseudo-biofeedback tasks and had no supplementary tasks during exposure. The tasks assigned to the patient groups are described below.

HRV biofeedback: The training session consists of verbal instructions to synchronize breathing with changes shown in heart rate (HR). All instructions are adapted from a well-established HRV biofeedback training provided by Lehrer et al., 2000 [23]. Participants are instructed to inhale when HR increases and exhale as it decreases.

Pseudo-biofeedback: The pseudo-biofeedback training also uses verbal instructions. Unlike in HRV biofeedback, participants are asked to synchronize a specific movement (tapping or a smooth, rhythmic hand movement) with the displayed signal (HR changes). At the end of the training sessions, all participants receive an MP3 file to continue their training at home. This file contains audio instructions for a 20 min session, during which each type of training (HRV and/or pseudo-biofeedback) is practiced for 10 min [16,24]. For this study, data from Groups 2 and 4 are considered, and five records from each group are used.

### 2.4. Electrocardiogram (EKG) Analysis and Construction of the Transition Matrix

EKG analysis is a method for diagnosing heart malfunction. The EKG signal consists of a smooth P wave, a QRS complex, and a T wave. Periods of time in the EKG, RR intervals, are defined by the duration from one R peak of the QRS complex to the next R peak of the next QRS complex. The duration of these intervals is known as heart rate variability (HRV) and is the change in duration of time measured between two adjacent heartbeats. Taking sufficiently long time intervals, HRV can be used as a noninvasive method to measure any aspect of cardiac activity. The duration of these intervals can range from a few seconds to minutes. In our case, the length of the intervals is approximately 6 to 12 s.

For this research, a classification parameter is established for our study groups. This parameter is defined as follows:

$$\alpha_{1}=\left[800\;\mathrm{ms},1000\;\mathrm{ms}\right]\;\mathrm{and}\;\alpha_{2}=\left[900\;\mathrm{ms},1100\;\mathrm{ms}\right]$$

for the arachnophobic and the hypertensive/normotensive groups, respectively, since, under neutral conditions, the average RR interval is about 900 ms [25]. Consequently, the $\alpha_{1}$ and $\alpha_{2}$ intervals would enable the observation of normotensive individuals with arachnophobia and hypertensive patients, respectively.

Before starting with the analysis and classification, for the processing of EKG signals and obtaining HRV, we used the QRSTool software (version 1.2) [26,27]. QRSTool is an open-source software available at www.psychofizz.org (accessed on 2 May 2024) and designed for analyzing cardiac signals, specifically electrocardiograms. This tool provides functions for both automatic and manual detection of QRS peaks besides calculating parameters such as the duration and amplitude of these waves. In the context of our study, we opted for manual detection, which helped us to conduct a detailed identification of each R peak while avoiding overlapping or missing points.

To construct the transition matrix, we define our state space *S* as follows:

S={A,N},

where *A* and *N* denote the arrhythmic and normal states, respectively. Thus, we obtain the following 2×2 transition matrix, *P*, given by the following:

$$P=\left[\begin{array}{cc} P_{AA} & P_{AN}\\ P_{NA} & P_{NN} \end{array}\right].$$

To calculate the probabilities $P_{ij}$, where i,j∈{A,N}, a classification algorithm built on Python 3.11.4. was proposed. This algorithm uses a text file containing the HRV information and generates a moving window that takes a certain number of RR intervals. The size of this window is adjustable, allowing for its modification to either decrease or increase its extent; in this case, the window size was set to include 10 RR intervals. The intervals contained within each window are categorized based on the two defined states (A or N), determined by their length and their compliance with the established α parameters. This process generates a sequence of arrays $V_{n}$, whose size depends on the size of the moving window and the EKG recording time. These arrays are presented as follows:

$$V_{n}=\left[A,A,A,N,A,N,\ldots \right].$$

Finally, the proposed algorithm analyzes the arrays $V_{n}$ by identifying the frequency at which a transition occurs from one state to another, thus obtaining the value of each of the probabilities $P_{ij}$, while verifying that the conditions hold.

$$P_{AA}+P_{AN}=1\;\mathrm{and}\;P_{NA}+P_{NN}=1$$

The probabilities are calculated for each $V_{n}$, providing information about the likelihood of a patient being in either a normal (N) or an arrhythmic (A) state at different moments throughout the EKG recording [28,29].

## 3. Results

### Markov Chain

Two groups of individuals with arachnophobia were compared in this study. In the group with techniques, participants utilized strategies to manage their reactions both before and during exposure to videos of spiders. Conversely, in the group with no techniques, participants employed these strategies only immediately before they were exposed to the videos.

**Figure 1:**
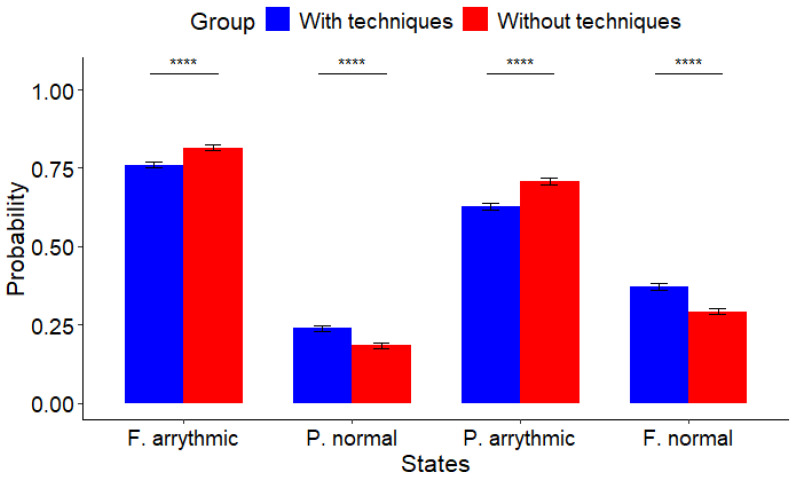
Comparison of two groups of individuals with arachnophobia. In Group 2 (with techniques), participants employed strategies to manage their reactions both before and during exposure to spider videos, while in Group 4 (without techniques), participants utilized the strategies solely before exposure to the videos. Data from Group 2 (blue color) indicate a smaller probability of experiencing arrhythmic states, with participants more frequently found in a normal state compared with those in Group 4 (red color) (**** *p* ≤ 0.0001).

Data indicate that participants in the group with techniques, shown in blue, exhibit a lower probability of experiencing an arrhythmic state and are more inclined to remain in a normal cardiac state in contrast with those in the group without techniques, displayed in red. See Figure 1 and Table 1 for visual and numerical details, respectively.

The initial bars depicted in Figure 1 represent the probability of a fully arrhythmic state (F. arrhythmic), indicating the transition from one arrhythmic to another arrhythmic state (A to A). Statistically significant differences (p=0.000002) were found between the group without techniques (0.784 ± 0.370) and the group with techniques (0.761 ± 0.366), as detailed in Table 1.

The probability of transitioning from a normal state to a fully normal cardiac state (F. normal) displayed a statistically significant difference (p=0.000062). Notably, higher values were reported in the group with techniques (0.372 ± 0.430) compared with the group without techniques (0.307±0.427), as illustrated in Figure 1 and Table 1.

The third set of bars shown in Figure 1 represents the partially arrhythmic state (P. arrhythmic); i.e., the transition from a normal to an arrhythmic state (N to A) is depicted, as detailed in Table 1. Comparing the two groups, a statistically significant difference (p=0.000062) was found between the group without techniques (0.693±0.430) and the group with techniques (0.628±0.427), as outlined in Table 1.

The final bars in Figure 1 illustrate the partially normal state (P. normal), indicating the transition from an arrhythmic state to a normal state, which is more pronounced for the group utilizing techniques (0.239±0.366) in comparison with the group utilizing no techniques (0.216±0.370). This difference was statistically significant (p=0.000002), as outlined in Table 1.

**Figure 2:**
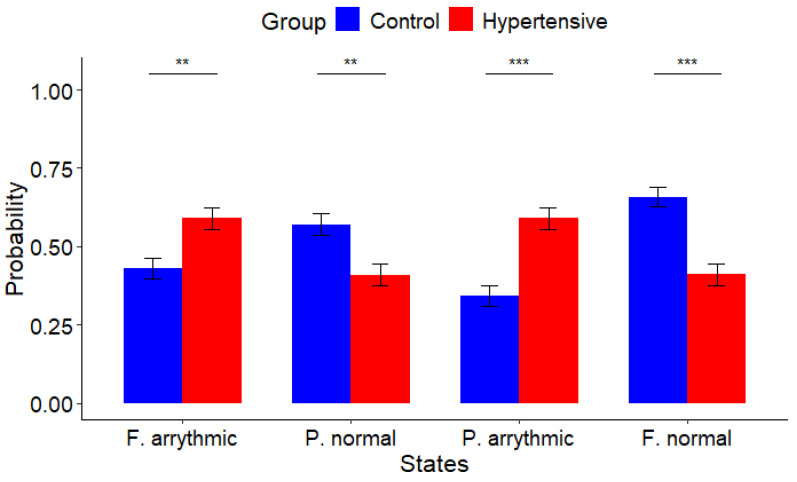
Probability comparisons per state transition. Observations revealed significant differences between hypertensive individuals, represented in red, and normotensive counterparts, represented in blue, indicating a higher probability of transition towards arrhythmic states among patients with hypertension (** *p* ≤ 0.01 and *** *p* ≤ 0.001).

The hypertensive group, represented in red, and the normotensive group, represented in blue, exhibit notable disparities, as evidenced in Figure 2 and Table 2. This observation reflects a quantitative and analytical distinction of heart functioning among participants.

As in the previous case of study, the first bars depicted in Figure 2 represent the fully arrhythmic state (F. arrhythmic), i.e., transitioning from one arrhythmic state to another (A to A). A statistically significant difference (p=0.00065) was found between hypertensive patients (0.591±0.478) and normotensive participants (0.430±0.438), as shown in Table 2.

The second set of bars in Figure 2 represents the transition from a normal to another normal state (F. normal). Comparing the normotensive group (0.658±0.408) and the hypertensive group (0.411±0.481), a statistically significant difference was reported (*p* = 0.00009), as detailed in Table 2.

In the third set of bars in Figure 2, representing the partially arrhythmic state (P. arrhythmic), i.e., the transition from a normal to an arrhythmic state (N to A), a statistically significant difference (p=0.00009) was also reported between the hypertensive group (0.589±0.481) and the normotensive group (0.342±0.408), as shown in Table 2.

Lastly, the final bars in Figure 2 illustrate the partially normal state (P. normal), i.e., indicating the transition from an arrhythmic state to a normal state. In this regard, a statistically significant difference (p=0.00065) was reported concerning the normotensive group (0.570±0.438) and the hypertensive group (0.409±0.478), as outlined in Table 2.

## 4. Discussion

Markov chains are useful tools for modeling epilepsy [1] and understanding the dynamics of cardiac electrical activity [29,30]. By dividing an EKG into discrete time segments, these chains can describe how the heart’s electrical patterns change from one state to another, facilitating the identification of abnormal patterns suggestive of cardiac arrhythmias. In several studies, such as the one conducted by Savorgnan et al., 2023 [30], Markov chains were used to forecast cardiovascular anomalies by analyzing 6 h EKG recordings per patient. In such analysis, a two-state Markov chain was proposed to identify cardiac arrest, attaining an accuracy of 82%. Similarly, for this study, a basic model based on Markov chains was proposed to determine the probability of a patient experiencing respiratory sinus arrhythmia (RSA) by analyzing 5 min EKG recordings per patient, which highlights the potential to characterize both experimental groups regarding hypertension and arachnophobia. This analysis utilizes EKG records of patients and aims to identify changes in RR interval measurements.

During the analysis conducted for the proposed groups, significant differences were found. These differences can be attributed to the detailed analysis of small time intervals across the records, providing more precise information about changes in HRV compared with analyzing the complete EKG.

The results showed more pronounced differences between individuals with hypertension and those with normal blood pressure, which might indicate that individuals outside the normal blood pressure range are more prone to arrhythmic states. This may be attributed to the effects of medications used to treat hypertension, which can impact heart rate and RR interval variability. A further exploration of these findings may provide valuable insights into the interplay between hypertension and cardiac rhythm disturbances as non-chaotic behavior [15].

Moreover, the comparison between hypertensive and normotensive groups unveiled significant differences in arrhythmic patterns. Hypertensive patients, using medications such as telmisartan and losartan, demonstrated a higher propensity for arrhythmic states and a lower probability of transitioning to normal cardiac states compared with normotensive participants. Detailed statistical analyses are provided in Figure 2 and Table 2.

Comparative analysis between the two groups of individuals with arachnophobia revealed noteworthy distinctions. Participants employing strategies to manage their reactions before and during exposure to spider videos exhibited a reduced probability of entering an arrhythmic state. Likewise, those participants employing the strategies were more likely to maintain a normal cardiac state compared with those without such techniques; see Figure 1 and Table 1 for details.

By extrapolating our findings on HRV in individuals with arachnophobia, a better understanding of fear and anxiety management might be achieved. HRV can be regarded as a biomarker that can be used to track changes in affective states, allowing early detection of the onset and relapse of depression, as outlined by Gullett et al., 2023 [31]. Therefore, our methodology could benefit clinical interventions by setting, in future works, a threshold for predicting when a patient with emotional disorders might transition into an arrhythmic state and taking medical action when this threshold is met.

Despite the promising results, the analysis remains basic. It is proposed to incorporate the recording of patients’ respiration in future research to enhance the accuracy of transition probability calculations and obtain more precise results, given that respiratory and cardiac oscillations are linked to each other [32]. Thus, breathing rhythm recordings might offer additional information regarding transitioning into an arrhythmic state, especially breath exhalation when the heart rate decreases [10]. In the future, we will aim to analyze RSA in psychiatric patients by monitoring their EKG (wearable) recordings to understand their behavior patterns and provide timely assistance when needed.

Furthermore, for future research, it is noteworthy that a significant portion of the existing literature on heart diseases, especially in analytical and diagnostic contexts, has historically emphasized the utilization of Poincaré maps, described in detail by Piskorski and Guzik, 2005 [33], and broadly used to quantitatively characterize patient groups by physiological time series recordings and their spatial distribution represented by the SD1 and SD2 descriptors, e.g., [34,35,36], and, more recently, novel methodologies such as those based on the Complex Correlation Measure (CCM) proposed by Karmakar et al., 2009 [37], and implemented as a complementary tool of Poincaré plots to understand and track temporal changes in time series from physiological records, e.g., [38,39,40]. Moreover, it is imperative to undertake a systematic and rigorous comparative analysis to identify the respective scopes and limitations of each framework. Such analysis would facilitate the identification of medical scenarios where each method proves most reliable.

Additionally, an examination of the sensitivity of each method could be conducted, given that a combination of visual and quantitative approaches remains prevalent in diagnosing heart conditions that might lead to undiagnosed or late-diagnosed heart conditions, especially those in early stages, where fluctuations in heart rate due to cardiac muscle damage may go largely un-noticed by medical personnel.

The proposed algorithm, utilizing Markov chains, was designed to discern alterations in heart rate variability to assess the probability of patients experiencing RSA. This investigation incorporated an analysis of EKG records from individuals with and without hypertension and individuals with arachnophobia exposed to videos of spiders and aided with and without techniques to face such situations.

Overall, our findings underscore the efficacy of the algorithm, particularly when applied over shorter time intervals, in discerning cardiac variability patterns associated with specific conditions or interventions, thus advancing our understanding of RSA dynamics. These insights hold potential implications for tailored diagnostic and therapeutic strategies, emphasizing the importance of optimizing analysis techniques for improved clinical outcomes.

However, the Markov chain methodology presented here relies on the accurate manual identification of R peaks, which can be time-consuming and subject to human error. Additionally, the methodology depends on external software for HRV measurement, which may introduce variability in results due to differences in software algorithms and processing techniques. These factors could potentially affect the accuracy of our method in medical scenarios.

## 5. Conclusions

The hypothesis that Markov chains provide a powerful and versatile method for analyzing RSA in cardiac muscle for both hypertensive patients and arachnophobic individuals was confirmed and supported by the statistical analyses conducted. Participants employing strategies to manage their reactions before and during exposure to spider videos exhibited a reduced probability of entering an arrhythmic state, and they were more likely to maintain a normal cardiac state compared with those without such techniques; this might be attributable to the emotional resilience developed by the use of the taught techniques. Hypertensive patients, using medications such as telmisartan and losartan, demonstrated a higher propensity for arrhythmic states and a lower probability of transitioning to normal cardiac states compared with normotensive participants.

By leveraging the inherent temporal dependencies in heart rate variability data, Markov chain models provide critical insights into autonomic regulation, cardiac function, and cardiovascular health, ultimately advancing our understanding of RSA dynamics and its clinical significance.

## Figures and Tables

**Table 1:** Statistical comparisons between the groups of individuals with arachnophobia. Transition probabilities were compared by conducting a Mann–Whitney U test of means due to the lack of data normality assumption.

Probability	*p*-Value	Mean		SD	
		**With Techniques**	**Without Techniques**	**With Techniques**	**Without Techniques**
A to N	0.000002	0.239	0.216	0.366	0.370
A to A	0.000002	0.761	0.784	0.366	0.370
N to A	0.000062	0.628	0.693	0.430	0.427
N to N	0.000062	0.372	0.307	0.430	0.427

**Table 2:** Statistical comparisons between the normotensive and hypertensive groups. Transition probabilities were compared by conducting a Mann–Whitney U test of means due to the lack of data normality assumption.

Probability	*p*-Value	Mean		SD	
		**Normotensive**	**Hypertensive**	**Normotensive**	**Hypertensive**
A to N	0.00065	0.570	0.409	0.438	0.478
A to A	0.00065	0.430	0.591	0.438	0.478
N to A	0.00009	0.342	0.589	0.408	0.481
N to N	0.00009	0.658	0.411	0.408	0.481

## Data Availability

The dataset utilized for the analysis of individuals with arachnophobia is sourced from records accessible within the Physionet databases, accessible through the following link: https://www.physionet.org/content/ecg-spider-clip/1.0.0/ (accessed on 2 May 2024). These data are provided in digital format, containing electrocardiogram (EKG) and respiration records in .txt format. Additionally, the complete code of the proposed algorithm is accessible via the following link: https://drive.google.com/drive/folders/17wP4TVUSjZudP6kdcYK51QT0RlPHUCIK?usp=drive_link (accessed on 2 May 2024). This repository also includes the inter-beat intervals (IBIs) of both hypertensive and normotensive individuals in .txt format.

## Abbreviations

The following abbreviations are used in this manuscript:

RSA	Respiratory Sinus Arrhythmia
BMI	body mass index
SBP	systolic blood pressure
DBP	diastolic blood pressure
HR	heart rate
EKG	electrocardiogram
HRV	heart rate variability
A	arrhythmic state.
N	normal state
A to N	probability of transitioning from an arrhythmic (A) state to a normal (N) state
A to A	probability of transitioning from an arrhythmic (A) state to an arrhythmic (A) state
N to A	probability of transitioning from a normal (N) state to an arrhythmic (A) state
N to N	probability of transitioning from a normal (N) state to a normal (N) state
